# 

**Published:** 1894-12

**Authors:** 


### TABLE OF CONTENTS.

ORIGINAL COMMUNICATIONS. PAGE

The Causation of Dental Erosion. By A. P. BRUBAKER, M.D., D.D S., Philadelphia . . . . . . . . 739

The Laboratory Method in Dental Education. By EDWARD C. KIRK, D.D.S., Philadelphia . . . . . . . . 746

Some Details as to the Care of Dental Instruments. By WILLIAM H. POTTER, D.M.D., Boston . . . . . . . . 754

Cervical Cavities and their Treatment. By WILLIAM L. FISH, D.D.S., Newark, N. J. . . . . . . . . 758

Is Prosthetic Dentistry lagging? No. By WILLIAM H. TRUEMAN, D.D.S., Philadelphia . . . . . . . . 761

REPORTS OF SOCIETY MEETINGS.

American Academy of Dental Science . . . . . . . . 768

Odontological Society of Pennsylvania . . . . . . . . 781

Harvard Odontological Society . . . . . . . . 786

EDITORIAL.

Thoughts, Past and Present . . . . . . . . 789

BIBLIOGRAPHY . . . . . . . . 793

FOREIGN CORRESPONDENCE.

London Letter . . . . . . . . 797

CURRENT NEWS.

Woman's Dental Association of the United States . . . . . . . . 801

Ohio State Dental Society . . . . . . . . 801

SELECTIONS.

Chemical Researches on the Mineral Matter of Bone and Teeth . . . . . . 802

